# Reconstruction of long‐term sublethal effects of warming on a temperate coral in a climate change hotspot

**DOI:** 10.1111/1365-2656.14225

**Published:** 2024-11-19

**Authors:** Marina J. Vergotti, Juan P. D'Olivo, Thomas C. Brachert, Pol Capdevila, Joaquim Garrabou, Cristina Linares, Philipp M. Spreter, Diego K. Kersting

**Affiliations:** ^1^ Institut für Geologische Wissenschaften Freie Universität Berlin Berlin Germany; ^2^ Unidad Académica de Sistemas Arrecifales Instituto de Ciencias del Mar y Limnología, Universidad Nacional Autónoma de México Puerto Morelos Mexico; ^3^ Institut für Erdsystemforschung und Fernerkundung, Universität Leipzig Leipzig Germany; ^4^ Departament de Biologia Evolutiva, Ecologia i Ciències Ambientals Facultat de Biologia, Universitat de Barcelona (UB) Barcelona Spain; ^5^ Institut de Recerca de la Biodiversitat (IRBIO), Universitat de Barcelona (UB) Barcelona Spain; ^6^ Institut de Ciències del Mar (ICM), Consejo Superior de Investigaciones Científicas (CSIC) Barcelona Spain; ^7^ Instituto de Acuicultura Torre de la Sal, Consejo Superior de Investigaciones Científicas (CSIC) Ribera de Cabanes Spain

**Keywords:** *Cladocora caespitosa*, marine heatwaves, Mediterranean Sea, paleo‐reconstruction, sclerochronology, sublethal effects, temperate coral

## Abstract

1. The impact of warming on zooxanthellate corals is widespread, from tropical to temperate seas, with its associated mortalities causing global concern.
2. The temperate coral *Cladocora caespitosa* is the only zooxanthellate coral with reef building capacity in the Mediterranean Sea, a climate change hotspot with warming rates triple the global average.
3. During the past two decades, *C. caespitosa* populations have suffered severe mortality events associated with marine heatwaves (MHWs). However, with monitoring efforts beginning, at best, in the 2000s, the occurrence of MHWs before that period, as well as the sublethal effects of these events remain poorly understood.
4. Here, we use sclerochronology to reconstruct the histories of past stress events and long‐term sublethal effects on *C. caespitosa* in three locations along a latitudinal gradient within the NW Mediterranean Sea, each with different environmental conditions.
5. Skeletal extension, density and calcification rates were compared with the in situ seawater temperature of each site to assess their relationship. Furthermore, we assessed the occurrence of skeletal growth anomalies to reconstruct stress events between 1991 and 2021, a period that encompasses the onset and evolution of warming‐related mass mortality events in the NW Mediterranean Sea.
6. Our results reveal a positive association between calcification and temperature, following a latitudinal temperature gradient. However, the evolution of the likelihood distribution of growth rates in the warmest site (Columbretes Islands) since the 1990s indicates a decrease in linear extension and calcification rates during the most recent years. With the increase in the frequency of MHWs and growth anomalies during the last decade, this decline suggests recurrent physiological stress events.
7. These results unravel information on the long‐term impacts of warming on coral growth and highlight the potential of applying sclerochronology to reconstruct the sublethal effects of warming using *C. caespitosa*.

## Graphical abstract

This study uniquely analyzes calcification in the temperate coral *Cladocora caespitosa* across unprecedented geographical and temporal scales in the Mediterranean Sea. By examining sublethal responses and comparing them to mortality data, we also offer a comprehensive understanding of coral responses to climate change impacts in the Northwestern Mediterranean Sea.

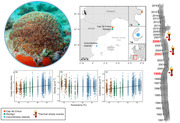

## 1 INTRODUCTION

Global climate change is warming the oceans, with the most optimistic scenarios predicting a rise in seawater temperature of at least +1°C by the end of the century (IPCC, 2019). Seawater warming is putting unprecedented pressure on benthic communities, increasing the mortality rates of a large number of key species (Garrabou et al., 2022; Hughes et al., 2018; Smale et al., 2019). The Mediterranean Sea is a climate change hotspot (Garrabou et al., 2022; Lejeusne et al., 2010), with warming rates over the last decade more than three times higher than the global average (IPCC, 2019). In the NW Mediterranean, extreme climatic events, such as marine heatwaves (MHWs), have increased in frequency since the early 2000s (Garrabou et al., 2022; IPCC, 2019; Martínez et al., 2023). Temperature during these MHWs can exceed physiological thermal limits triggering mass mortality events (MMEs) across different taxa (Garrabou et al., 2009, 2022; Marbà et al., 2015). These events mainly affect long lived species, with slow population dynamics and bioengineering capacity (Garrabou et al., 2009, 2022; Grenier et al., 2023; Kersting et al., 2013), causing irreversible structural changes in the functional assemblages of benthic communities (Garrabou et al., 2022; Gómez‐Gras, Linares, Dornelas, et al., 2021 Gómez‐Gras, Linares, López‐Sanz, et al., 2021; Verdura et al., 2019).

The widespread lethal impact of warming on Mediterranean benthic organisms particularly affects hexa‐ and octocorals (Garrabou et al., 2019; Gómez‐Gras, Linares, López‐Sanz, et al., 2021; Jiménez et al., 2016; Kersting et al., 2013; Kružić et al., 2014; Verdura et al., 2019). However, the sublethal effects of warming on these organisms are poorly understood. Most studies exploring this topic have focused on the Mediterranean gorgonians, revealing that repeated thermal stress negatively impacts their reproduction cycle and alters their phenology (Kipson et al., 2012; Linares et al., 2008; Viladrich et al., 2022). Such results illustrate the vulnerability of Mediterranean foundational species beyond mortality, hindering their potential to recover from extreme climatic events. However, to our knowledge, no studies have delved into the long‐term sublethal effects of rising temperatures and MHWs on other Mediterranean foundational species such as corals.

The Mediterranean endemic scleractinian coral *Cladocora caespitosa* has been affected by climate change, experiencing recurrent mortality events caused by warming (Jiménez et al., 2016; Kersting et al., 2013; Rodolfo‐Metalpa et al., 2005). As the only Mediterranean zooxanthellate coral with reef‐building capacity, *C. caespitosa* is a key bioengineering organism in the region, providing shelter and habitat to many species (Kersting & Linares, 2012; Kružić & Benković, 2008; Peirano et al., 1998, 2001; Pitacco et al., 2017). The good adaptation of this coral to the high seasonality of the Mediterranean allows it to grow in a wide range of environmental conditions (Hoogenboom et al., 2010; Peirano et al., 1998; Schiller, 1993; Zibrowius, 1980). Indeed, *C. caespitosa* can thrive both in well‐lit and dim‐light conditions, thus occurring in shallow photophilic communities, often within algal assemblages (Kersting et al., 2017, 2023; Kersting & Linares, 2012; Pons‐Fita et al., 2020, 2021), but also in mesophotic communities (Kersting et al., 2015; Morri et al., 1994). Due to its slow life history (Kersting, Teixidó, & Linares, 2014) and the increasing impacts of climate change, this species was included as endangered on the IUCN Red List (Casado‐Amezúa et al., 2015; Kersting et al., 2022). Understanding the sublethal effects of warming on *C. caespitosa* skeletal growth is essential to assess their health, predicting population trends and planning management and conservation strategies.

Sclerochronology, the study of physical and chemical variations in the accretionary hard tissues, is a key method to study the environmental change recorded by coral skeletons. For *C. caespitosa*, environmental changes, primarily related to seasonality, lead to the yearly deposition of a high‐density (HD) band in winter and a low‐density (LD) band in summer that can be revealed using X‐rays (Peirano et al., 1999). Using sclerochronology, seasonal and annual growth parameters (linear extension rate, calcification rate and bulk density) can be calculated from those bands and used to reconstruct the past life history of the coral and the evolution of its growth under distinct environmental conditions (Highsmith, 1979; Kersting & Linares, 2012; Kružić et al., 2012; Morri et al., 2001; Peirano et al., 1999; see Lough & Cooper, 2011 for a review). In addition, the occurrence of growth anomalies, such as stress marks, allows reconstructing periods of acute stress (Barkley et al., 2018; DeCarlo et al., 2019; Kersting & Linares, 2019).

This study provides, for the first time, a long‐term analysis of the sublethal impacts of warming and MHWs on Mediterranean corals. We reconstruct the growth histories of *C. caespitosa* populations in three sites of the NW Mediterranean Sea over 10 and 23 years, coinciding with increased warming impacts in the region (Garrabou et al., 2009, 2022) providing baseline growth data across geographical and bathymetric gradients.

## 2 MATERIALS AND METHODS

### 2.1 Coral sampling and environmental data

**FIGURE 1:**
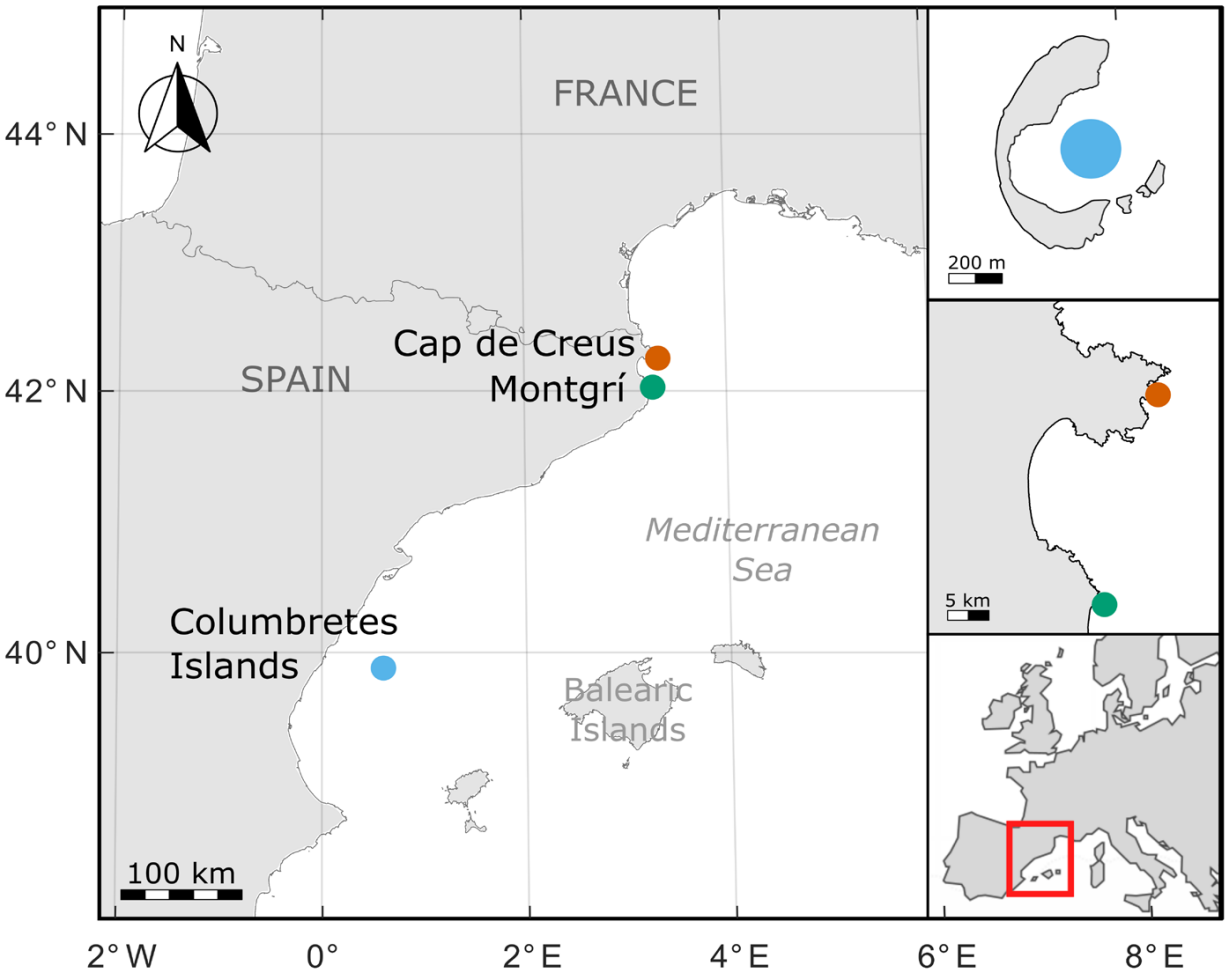
Location of study sites along the northeast coast of Spain: Columbretes Islands (blue), Montgrí (green), and Cap de Creus (orange). Map lines delineate study areas, not necessarily country boundaries.

Corallites from living colonies of the *C. caespitosa* coral were collected from three sites along the Spanish coast of the NW Mediterranean Sea (Figure 1). The number of colonies and corallites analysed at each site is as follows (from south to north): Columbretes Islands 230 corallites from 12 colonies, Montgrí 105 corallites from eight colonies, and Cap de Creus 70 corallites from nine colonies. In the Columbretes Islands, samples were collected annually in August between 2015 and 2018, and again in October 2022, and in Cap de Creus and Montgrí in September 2020. This article did not require ethical approval from an animal welfare committee.

Daily surface temperature (SST) data from 1 m depth have been collected in the bay of Illa Grossa (Columbretes Islands) since 1991 using a mercury‐in‐glass thermometer, and summer SST anomalies were obtained between 1991 and 2012 (Kersting et al., 2013). In situ temperature at 15 m depth (exactly where the *C. caespitosa* population is located) has been recorded hourly in the Columbretes Islands with an autonomous sensor (HOBO v2 Water Temp Pro sensor) since 2008. A significant warming trend was found in both datasets between 1991 and 2012 for SST (Kersting et al., 2013), and between 2007 and 2019 for in situ temperature (Martínez et al., 2022). For Montgrí and Cap de Creus, we used in situ temperatures recorded at 20 and 25 m, respectively (T‐MEDNet, https://t‐mednet.org/), corresponding to the depths where the coral colonies grow. Annual, summer and warmest month averages were obtained for all temperature products.

### 2.2 X‐ray radiograph analyses and sclerochronology

The colony fragments were cleaned of organic matter and debris in 1:1 NaOCl and deionized water solution for 24 h (Nagtegaal et al., 2012), rinsed with deionized water and left to dry at room temperature for 24 h. Individual corallites were separated using a mechanical handheld saw, also removing calcareous algae and shell remnants, after which dust and particles were blown off with compressed air and damaged corallites were discarded. Positive X‐ray images of all selected corallites were obtained at the Institut für Erdsystemforschung und Fernerkundung (Leipzig) by exposing the samples for 60 s at a 44 kV voltage and 1000 μA intensity using a digital X‐ray cabinet. X‐ray images showing unclear growth bands, due to factors such as bioerosion, were discarded from further analysis. Following this screening process, X‐ray images of 230 corallites from the Columbretes Islands, 105 from Montgrí, and 70 from Cap de Creus remained suitable for analyses.

The selected X‐ray images were analysed using Coral XDS software (https://hcas.nova.edu/tools‐and‐resources/coralxds/index.html; Kersting & Linares, 2012; Kružić & Benković, 2008; Peirano et al., 1998, 2001; Spreter et al., 2022). A calibration procedure was followed, based on the thickness of the corallite samples, to convert X‐ray grey‐scale variations to bulk density (g CaCO_3_ ·cm^−2^ ·year^−1^, hereafter referred to as density) (see Method S1). Subsequently, the corallites were analysed by tracing transects along their length and avoiding the lateral edges and columella structures of the corallite (Figure S1). The linear extension rate (mm·year^−1^) was measured as the length of couplets of LD and HD growth bands, using the Half‐Range method of the software. The calcification rate (g CaCO_3_ ·cm^−2^ ·year^−1^) was obtained as the product of linear extension rate and density.

The age model for each corallite was established by counting annual density bands (couplets of LD and HD bands) backwards starting from their collection year. To avoid reporting incomplete years from bands still forming, counting began with the first complete annual band near the calix. Annual growth parameters were calculated as follows: linear extension as the sum of the LD and HD bands lengths for a given year, and calcification rate and density as the average of the corresponding seasonal values. The LD and HD band ratio (i.e. LD/HD) was also calculated.

Corallites and their X‐ray images were inspected for indications of anomalous growth, quantified per year and site. Anomalous growth marks were defined as alterations in the shape and skeletal structure of the corallites, often paired with a decrease in band density or linear extension (see Figure 5a and Kersting & Linares, 2019 for examples).

### 2.3 Statistical analyses

The average annual growth rates per site and associated standard deviation (SD) were calculated using the growth values of annual bands of all corallites. The average growth rates per site were then calculated over the common period between sites (2009–2019). The SD for the growth rates per site was estimated by propagating the SD of the average annual growth rates, following the error propagation method used in Vergotti et al. (2019): $\mathrm{\delta y}=\sqrt{\sum {\left(\delta x_{i}\right)}^{2}}$, where *δ* is the SD, *y* is the average growth rate per site, and *x*_*i*_ is a given average annual growth rate. Differences between sites over the common period were assessed with ANOVA tests using the average annual growth values of each site. Tukey's honestly significant difference (HSD) test was performed at *p* < 0.05 for multiple comparisons between sites, to determine whether they could be grouped.

To combine records of differing lengths and average values, a normalization process is required to ensure that all portions of the chronology are equally weighted. This normalization process allows us to properly study trends and relationships with environmental factors otherwise biased by individual variability in records of differing length (Dodge & Lang, 1983; D'Olivo et al., 2013; Manzello et al., 2021). Therefore, for each site separately, the annual growth parameters of each corallite were normalized by subtracting the corallites mean growth rates and adding the overall average growth values of the record (see Method S2).

The effect of seawater temperature on growth was tested using a set of linear mixed models (LMM). Three different models were fitted using the different normalized annual growth values (linear, density, and calcification) per corallite, and using the average seawater annual temperature, summer temperature, and temperature of the warmest month for each site as explanatory variables. To account for the lack of independence of growth for each corallite, a unique identifier was used for each corallite nested with site. The LMM were fitted using the “lmer” function from the “lme4” R package (Bates et al., 2014). Years with less than 5% of the total sample size of growth data of each site were excluded from the models, limiting the study periods to 1998 to 2021 for the Columbretes Islands, and 2010 to 2019 for Montgrí and Cap de Creus. Outliers were detected using the *z*‐score formula: $Z=\left|\frac{x-\mu }{\sigma }\right|$, where *x* represents a specific growth value, *σ* represents the standard deviation of the normalized annual growth values per corallite and *μ* represents the mean of the normalized annual growth values per corallite. Growth values with a *z*‐score of 3 and above were identified as outliers and removed before the test. Finally, the significance of seawater temperature on growth was tested by performing a Type III analysis of variance (ANOVA) using Satterthwaite's method to approximate the degrees of freedom, with the function ‘anova’ from the “lmerTest” R package (Kuznetsova et al., 2017). This approach tests the contribution of temperature while controlling for all other factors in the model, providing marginal *F*‐tests and corresponding *p*‐values for each fixed effect. To determine whether the relationship between growth and seawater average temperature was not lineal, the above described model structure was replicated using a generalized additive mixed model (GAMM) from the ‘mgcv’ R package (Wood, 2011), and setting an adaptive smoothing spline of seawater temperature as the explanatory variable. All models were tested for normality, dispersion and homoscedasticity (see Table S1). The LMM were also fitted separately for each site. In the case of the Columbretes Islands, the relationship with growth was tested against both the in‐situ temperature and SST, over the overlapping periods of both records (2008–2021).

Long‐term changes in growth rates in the Columbretes Islands were described using probability density functions (PDF), which indicate the likelihood of a variable to take on a particular value. PDFs were calculated using normalized bulk growth data over three periods: 1998 to 2002, 2003 to 2012, and 2013 to 2021. These periods were based on the reported increase in thermal stress and mortality events following 2003, for *C. caespitosa*, in the Columbretes Islands (Kersting et al., 2013). Here, we extend this knowledge beyond 2012 by examining the period from 2013 to 2021. PDFs were calculated following a normal distribution formula: $f\left(x\right)=\frac{1}{\sigma \sqrt{2\pi }}\cdot e^{\frac{\left(x-\mu^{2}\right)}{2\sigma^{2}}}$, where *x* represents a specific growth value, *σ* represents the SD of the growth record and *μ* represents its mean. The goodness of fit of the normal distribution of the growth was evaluated using the “fitdistrplus” R package (Delignette‐Muller & Dutang, 2015).

## 3 RESULTS

### 3.1 Growth characteristics of *Cladocora caespitosa* in the NW Mediterranean Sea and its relationship with temperature

The Columbretes Islands had the highest linear extension (3.20 ± 1.15 mm·year^−1^) and calcification rates (0.22 ± 0.09 g·cm^−2^), while Montgrí had the lowest density values (1.32 ± 0.32 g·cm^−3^; see Table 1). The bulk average LD/HD band ratio was slightly above 1 in all sites (Table 1), indicating that growth rates in summer and winter are very similar. Significant differences were found between sites for linear extension, density, and calcification (*F*_2,30_ > 3.403, *p* < 0.001), but not for the LD/HD band ratio values (*F*_2,30_ = 0.361, *p* = 0.700; Figure S2).

**TABLE 1:** Ranges of annual growth rates along with average annual growth rates ± SD calculated from annual growth bands for linear extension, density, and calcification, as well as LD/HD band extension ratio for each site.

	Linear extension (mm)	Bulk density (g·cm^−3^)	Calcification (g·cm^−2^)	Extension ratio LD/HD bands
Columbretes Islands	[2.93–3.39] 3.20 ± 1.15	[1.33–1.40] 1.36 ± 0.23	[0.20–0.23] 0.22 ± 0.09	[1.07–1.37] 1.22 ± 0.82
Montgrí	[2.29–2.90] 2.59 ± 0.88	[1.23–1.50] 1.32 ± 0.32	[0.15–0.19] 0.17 ± 0.07	[0.91–1.37] 1.15 ± 0.76
Cap de creus	[1.56–2.97] 2.39 ± 0.98	[1.39–1.64] 1.48 ± 0.24	[0.12–0.21] 0.18 ± 0.08	[0.57–1.66] 1.17 ± 0.67

*No*te: The values were obtained for the common period between sites (2009–2019).

**FIGURE 2:**
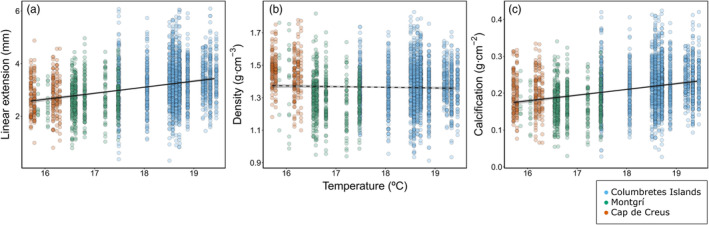
Average annual (a) linear extension, (b) density, and (c) calcification as a function of in situ temperature for all three sites. Black curves display the fit of the linear mixed model (LMM, in black) for the combined data from all sites, with the 95% confidence interval (in grey). Dashed lines indicate non‐significant relationships. Growth values from individual corallites, indicated by coloured points, are grouped by sites, i.e. Columbretes islands (in blue), Montgrí (in green), and Cap de Creus (in orange).

At the regional level (all sites), average annual linear extension and calcification were positively associated with average annual in situ temperature (Figure 2a,c), while the GAMMs did not obviously differ from a linear relationship (Figure S3). Similar relationships were found with summer average in situ temperature, and average in situ temperature of the warmest month (Table S2). For the Columbretes Islands, linear extension and calcification were positively associated with annual in situ temperature and SST, and in situ average summer temperatures (Table S2). Calcification was also positively associated with summer average SST, while density was positively associated with in situ temperatures and SST of the warmest month, and average summer SST (Table S2). For Montgrí and Cap de Creus, only density was negatively associated with all temperatures, except with in situ average temperatures of the warmest month for Montgrí. In the case of Montgrí, linear extension was also positively associated with average annual in situ temperatures.

### 3.2 Long‐term changes in growth rates at the population level

**FIGURE 3:**
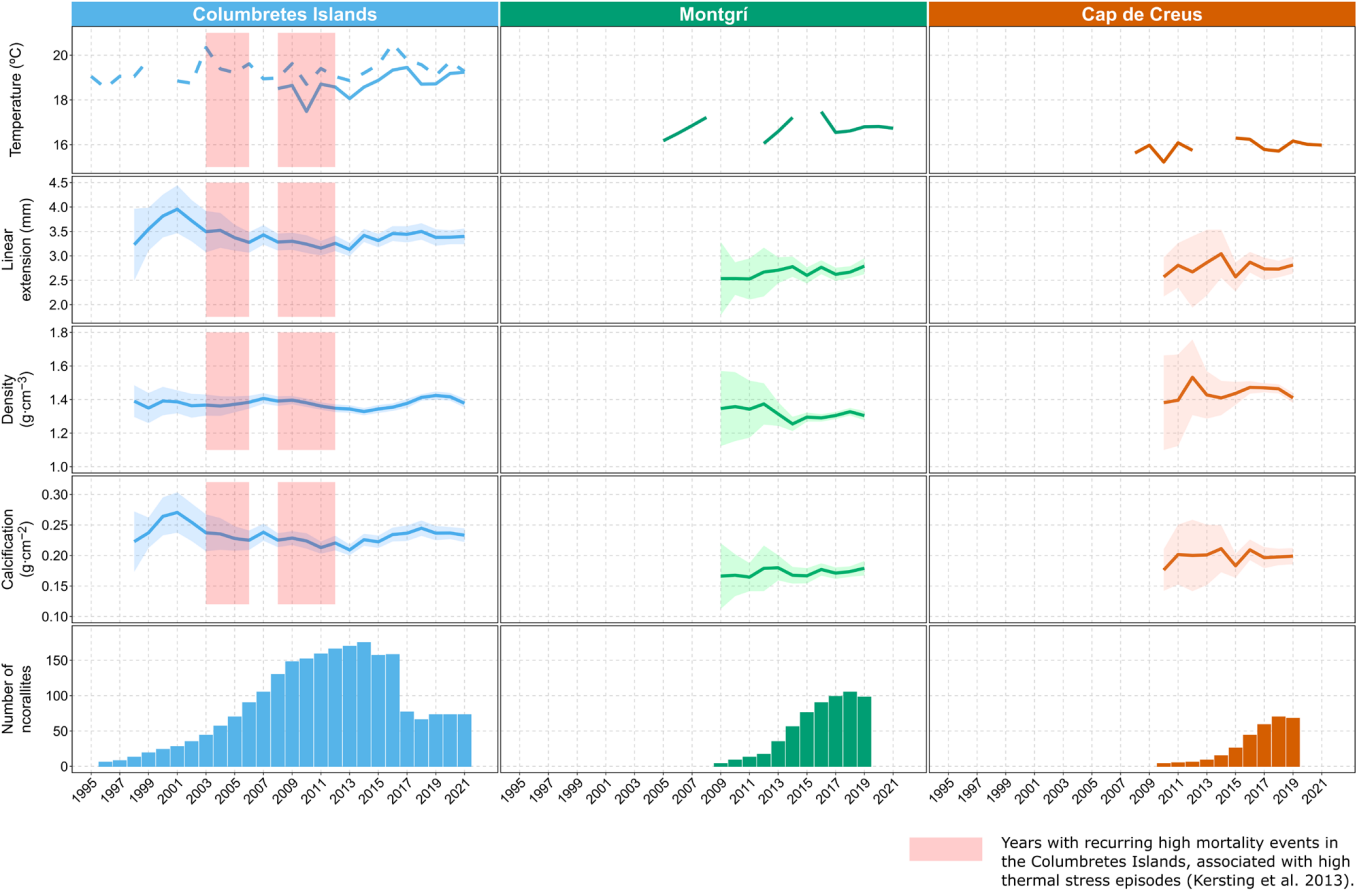
Temporal evolution of Sea Surface Temperature (SST, in dashed lines), in situ temperature (in full lines), and of the scaled average annual growth parameters of *Cladocora caespitosa* (linear extension, skeletal density, and calcification) together with their respective confidence intervals at 95%. These values are represented for each site: Columbretes islands (left, blue), Montgrí (center, green) and Cap de Creus (right, orange). Red‐shaded areas highlight years of recurring high mortality events in the Columbretes Islands, associated with high thermal stress episodes (Kersting et al., 2013).

When examining the long‐term changes in growth rates per site, the Columbretes Islands presented a negative trend in linear extension (*r* ^2^ = 0.717, *p* = 0.002) and calcification (*r* ^2^ = 0.595, *p* = 0.009) between 2003 and 2012, and a positive trend in density (*r* ^2^ = 0.635, *p* = 0.010) and calcification (*r* ^2^ = 0.536, *p* = 0.025) between 2013 and 2021. Montgrí had a significant increase in linear extension (*r* ^2^ = 0.441, *p* = 0.026) between 2009 and 2019, but no trend was observed for density or any of the growth parameters from Cap de Creus between 2010 and 2019 (Figure 3).

**FIGURE 4:**
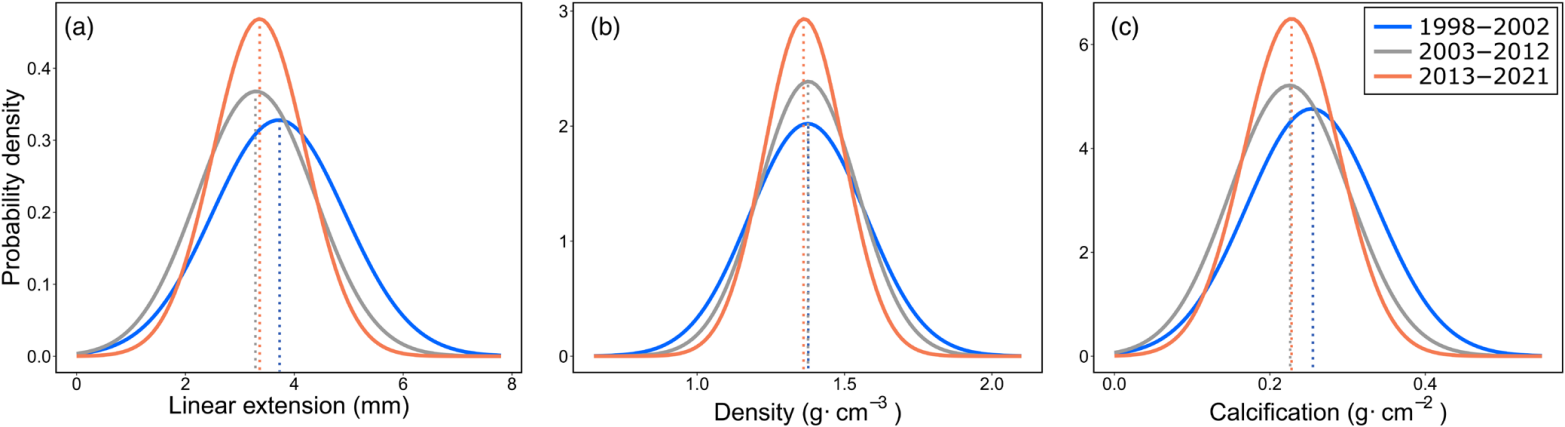
Probability density functions (PDFs) of three periods: 1998–2002 (blue), 2003–2012 (grey) and 2013–2021 (orange), represented for linear extension (a), density (b), and calcification (c) in corallites from the Columbretes Islands.

Long‐term changes in the growth rates of the Columbretes Islands corals were further analysed by comparing their PDFs over the three periods previously described (1998 to 2002, 2003 to 2012, and 2013 to 2021). The PDFs of linear extension and calcification showed a decrease in mean growth from the period 1998 to 2002 to the period 2013 to 2021, although this trend was not reflected in the density (Figure 4).

### 3.3 Long‐term quantification of growth anomalies

**FIGURE 5:**
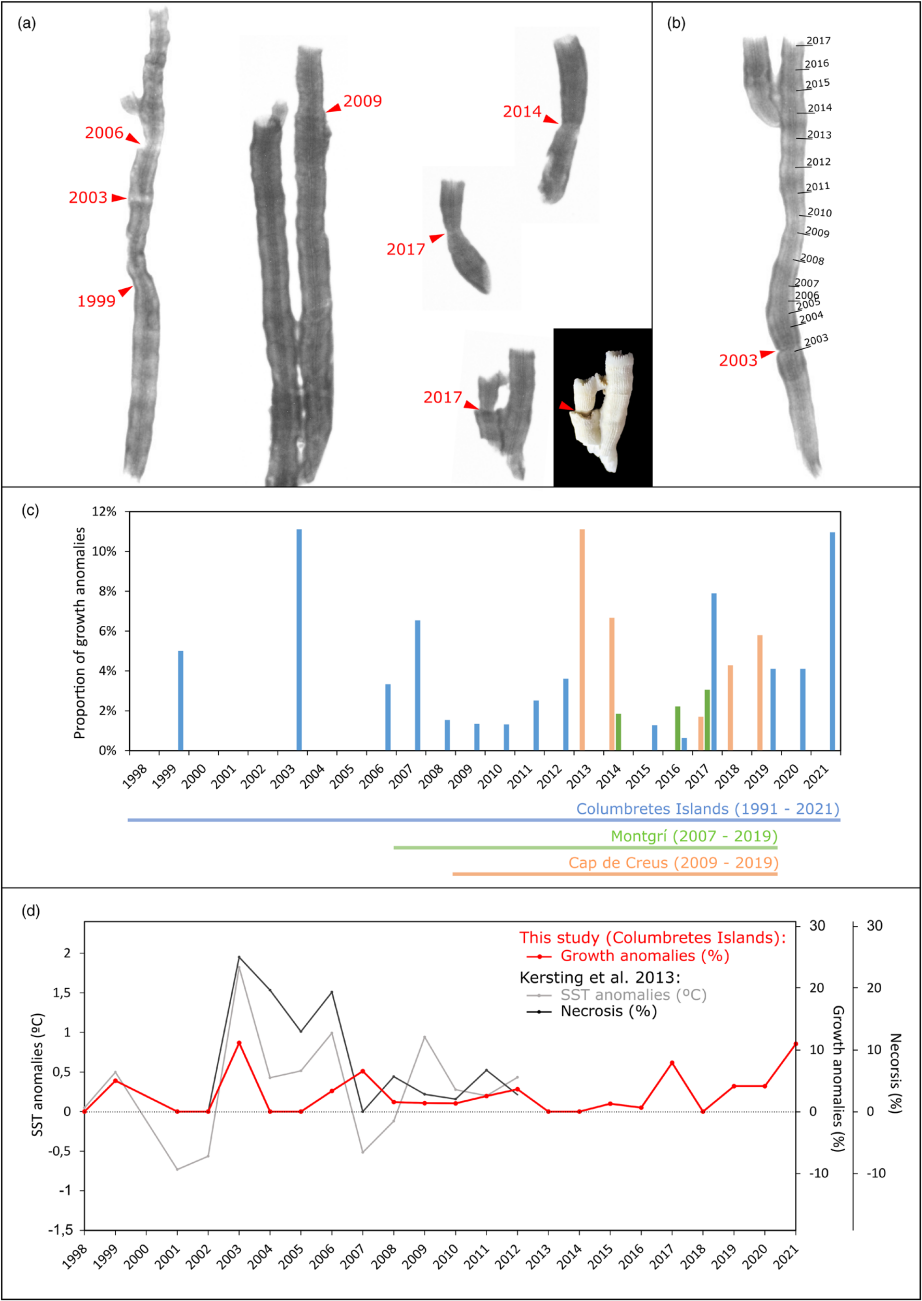
(a) Positive X‐ray images of *Cladocora caespitosa* corallites from all sites, showing a variety of growth anomalies with their corresponding year. (b) The timing of the growth anomalies was determined from the age model of the corallites. (c) Growth anomalies were quantified per year for the Columbretes Islands (in blue), Montgrí (in green), and Cap de Creus (in orange) using all the corallites available for these years. The number of growth anomalies is represented proportionally to the annual sample size at each site. The total period covered in each site is indicated under the plot with the coloured horizontal lines. (d) The proportion of corallites with growth anomalies per year in the Columbretes Islands compared to the proportion of necrosis per year and summer SST anomalies reported at the same site by Kersting et al. (2013).

Growth anomalies in the Columbretes Islands occurred annually between 2006 and 2012, with the highest number of growth anomalies observed per year in 2003 and 2021, followed to a lesser extent by 1999, 2007, and 2017 (Figure 5). Cap de Creus presented the highest number of growth anomalies in 2013, with proportions comparable to 2003 and 2021 in the Columbretes Islands. No other site presented anomalies during 2013. Montgrí presented the lowest proportions of growth anomalies of any site, concentrated in 2014, 2016, and 2017. Considering the common period for all three sites (2009–2019), growth anomalies were observed in all sites only in 2017. While the quantification of anomalous growth marks per year indicated notable differences between sites, it is important to underline the difference in the overall sample size and the number of years covered (Figure 5).

## 4 DISCUSSION

Marine heatwaves (MHWs) are occurring with increasing frequency and intensity in the Mediterranean Sea and are directly responsible for extensive mass mortality events (MMEs) (Garrabou et al., 2009, 2022). The Mediterranean coral *C. caespitosa* has been highly impacted by these events (Kersting et al., 2013; Kružić et al., 2014; Rodolfo‐Metalpa et al., 2005), and while the lethal impacts of MHWs on this coral have received significant attention, their sublethal effects remain understudied. With this study, we show the long‐term effects of temperature on the growth of the temperate zooxanthellate coral *C. caespitosa* along an environmental gradient across three sites in the NW Mediterranean Sea. We highlight the negative impacts of long interannual periods of continuous warming on the growth, and contribute with new insights to assist in the reconstruction of stress periods.

### 4.1 Effects of temperature on coral growth

Linear extension rates measured at the three locations of this study are within the range of values previously reported in the literature (see Table 1 in Kersting & Linares, 2012). They are also well below most of the major tropical reef‐building coral species (Highsmith, 1979). Additionally, the LD/HD band ratio close to 1 in all sites shows that growth rates during summer (high temperature, high light intensity) and winter (low temperature, low light intensity) are similar and the seasonal changes in growth were proportional between sites, despite different environmental settings. Similar results were found in the Balearic Islands (NW, Mediterranean, Kersting et al., 2023), indicating favourable environmental conditions for growth throughout the year in these sites, and highlighting the capacity of *C. caespitosa* to regulate its biomineralization despite the marked seasonality of the Mediterranean Sea. However, these results contrast with reports of slowed, or even arrested growth in other areas of the Mediterranean Sea where temperatures can fall below 11°C during winter (Kružić & Benković, 2008; Montagna et al., 2007; Peirano et al., 2009), indicating that extreme low temperatures can negatively impact coral growth. These results also show a major difference with most tropical corals that commonly have higher LD/HD ratio values, indicating that the growth rates of LD bands are greater than those of HD bands (Carricart‐Ganivet et al., 2000; Highsmith, 1979).

The northernmost populations of *C. caespitosa* in this study, that is Montgrí and Cap de Creus, exhibited, on average, slower growth compared to the Columbretes Islands. This difference can be attributed to a consistently higher water temperature throughout the year in the Columbretes Islands. When considering the populations of all three sites together, linear extension and calcification followed a positive linear relationship with average annual in situ temperature, while this relationship was non‐existent in the case of density. At the regional level, this result indicates a sensitivity to the thermal geographical gradient, with coral growth enhanced by warmer temperatures. Similar relationships have been obtained with SST for the same species in the Ligurian Sea (Peirano et al., 2004) and in the Adriatic Sea (Kružić et al., 2012), as well as in corals from tropical regions (e.g. Lough & Barnes, 2000; Manzello et al., 2021). However, the temperature‐growth response of corals has been described to follow a parabolic curve, indicating that corals can thrive under moderate to high temperatures but that they will flounder once a thermal threshold is crossed (Castillo et al., 2014; Edmunds, 2005; Peirano et al., 2009). Using aquaria experiments Rodolfo‐Metalpa et al. (2006) indicate that growth rates of *C. caespitosa* decrease past a thermal optimum of ~24°C. Nevertheless, our results, did not show a notable difference from a linear model, indicating at first sight that a thermal threshold may have not yet been reached in these populations. Further studies on coral growth in areas encompassing a wider range of temperature regimes are necessary to accurately determine the nature of this relationship at the extremes (i.e. low and high temperatures).

### 4.2 Long‐term growth responses along environmental gradients

The lack of long‐term growth trend found in Cap de Creus during the studied period and the significant increase found in linear extension and calcification in Montgrí, indicate no slowdown in their growth as a response to warming. Considering that both sites have markedly lower temperatures compared to the Columbretes Islands, these results could indicate that the coral colonies of Montgrí and Cap de Creus are still growing within their thermal limits. Nevertheless, the relative shortness of these records prevents a more in‐depth analysis. The Columbretes Islands had the longest record, allowing us to reconstruct growth rates back to the 1990s. This record revealed a high long‐term variability that can be separated into three periods. The first period (1998–2002) is characterized by the highest linear extension and calcification values, which then transited steeply towards lower values, reaching a minimum in 2003. The summer of 2003 recorded the strongest MHW of the decade in the Mediterranean basin, also triggering a widespread MME (Garrabou et al., 2009, 2022; Kersting et al., 2013). During the second period (2003–2012), both linear extension and calcification maintained a decreasing trend, coinciding with recurrent episodes of coral mortality and thermal anomalies reported between 2003 and 2006, and, to a lesser extent, between 2008 and 2012 (Kersting et al., 2013). These results contrast with those obtained by Kružić et al. (2012) in the Adriatic Sea, where a positive trend was found for corallite growth between 1987 and 2012. However, while the mean annual temperatures for that region were reported to stay below 20°C, daily summer maximum temperatures reached 29°C or more and were associated with high levels of bleaching and mortality (Kružić et al., 2014). During the last period (2013–2021), linear extension stabilized and density and calcification increased, following an apparent trend of recovery, despite the increasing frequency of MHWs in the NW Mediterranean (Garrabou et al., 2022; Martínez et al., 2022). However, the shift towards lower values in both linear extension and calcification after the period of 1998–2002 indicated by the PDF assessment casts doubt on this apparent recovery.

In the three populations, density showed more sustained stability over time compared to linear extension and calcification, with no association with temperature at the regional level. Similar relationships reported by Lough and Barnes (2000) for tropical corals indicate a reduced sensitivity of density to temperature variations. This may be the result of a growth strategy involving a trade‐off between extension and density, with calcification resources allocated to linear extension rather than density, a hypothesis already posited for tropical corals of the Great Barrier Reef (Carricart‐Ganivet, 2007; Carricart‐Ganivet et al., 2012). Thus, temperatures rising above a thermal threshold can increase the energy costs of survival, eventually causing decreases in calcification rates as a result of decreased linear extension rates while density remains stable. For corals benefiting from an added energy source, it would be possible to compensate for some of these costs and maintain their calcification. For example, Montgrí is the only site with a positive trend in calcification and linear extension. Due to its proximity to Cap de Creus and the resulting similar environmental setting, similar growth rates and trends would have been expected for both sites. However, Montgrí is also located close to the mouth of the Ter River, resulting in high nutrient inputs in the area (Ribes et al., 1999). As previously hypothesized, *C. caespitosa* colonies could benefit from these nutrient‐enriched conditions, possibly resulting in an increased resilience to summer heat stress (Kersting et al., 2023). Therefore, these conditions could also help *C. caespitosa* colonies overcome energy constraints that have been associated with warming‐related mortalities (Coma et al., 2009). The higher promotion of linear extension versus density that occurs in Montgrí due to this growth strategy is also consistent with the lower density levels compared to Cap de Creus, an outcome that was reported similarly for tropical corals by Risk and Edinger (2011). Further, while density was negatively associated with temperature in the case of Montgrí and Cap de Creus, this relationship was absent at the regional level, indicative of other non‐temperature related, site specific factors modulating density differently in each site. These findings again highlight similarities between temperate and tropical corals, pointing to some generalized responses across the Scleractinian order to changes in the environment.

### 4.3 Reconstructing stress events from coral skeletons

Sublethal evidence of physiological stress, manifested as growth anomalies, was found in corallites from all sites. The earliest evidence of growth anomalies, also referred to as stress marks, was found in the Columbretes Islands during 1999, when the first MME affecting benthic invertebrates over a large geographic scale in the NW Mediterranean was reported (Cerrano et al., 2000; Perez et al., 2000). However, prior to this study, no evidence was found indicating that the *C. caespitosa* population of the Columbretes Islands had been affected in some way by this event (Kersting et al., 2013). The magnitude and negative impact of the subsequent 2003 MHW are consistent with the highest occurrence of stress marks observed in the corallites of the Columbretes Islands, coinciding with the highest levels of necrosis reported to date in the area (Kersting et al., 2013). Between 2006 and 2012, a period characterized by recurrent warming‐related mortality events in the Columbretes Islands (Kersting et al., 2013), growth anomalies were detected yearly. The prevalence of these growth anomalies has reached particularly high levels in the last decade. Notably, the years 2017 and 2021 stand out, with the latter exhibiting anomalies akin to those recorded in 2003. This pattern is consistent with the progressive decline in linear extension and calcification rates depicted by the PDFs and casts further doubts on the potential recovery trend presented by growth rates in the later years of the record.

Some discrepancies were found between the percentages of growth anomalies and the incidence of necrosis reported for the Columbretes Islands. For instance, the summers of 2004 and 2005 were characterized by high mortality rates (Kersting et al., 2013), despite relatively mild positive thermal anomalies. This was hypothesized to be related to delayed physiological stress following the MHW of 2003 (Kersting et al., 2013). Although stress marks would have been expected during these years, none were detected in our analysis. The reason for this discrepancy is not clear. Furthermore, while 2007 had no indications of mortality and was the only year between 2002 and 2012 with a negative summer thermal anomaly (Kersting et al., 2013), the corallites of the Columbretes Islands presented high percentages of stress marks, which could be an indication of another non‐temperature related stress factor negatively impacting the population at the sublethal level. In this sense, it is important to note that the invasive algae *Lophocladia trichoclados* was first detected in the Columbretes Islands in 2006 and was reported to have rapidly invaded the hard substrate of the bay that harbours the *C. caespitosa* population, resulting in negative interactions with several local benthic species (Kersting, Ballesteros, et al., 2014; Kersting & García‐March, 2017). Although *L. trichoclados* does not grow directly on the coral colonies (Kersting, Ballesteros, et al., 2014) it has a shading effect that could reduce the energy obtained from respiration and feeding of symbionts and polyps possibly increasing the susceptibility of these corals to temperature (Kersting et al., 2015).

Montgrí and Cap de Creus only presented anomalies during the last decade, although the shortness of records for Cap de Creus limits this assessment. Stress marks appeared in the corallites of all three sites only in 2017, reported as the sixth warmest year since 1982, with a particularly long and strong MHW affecting the western Mediterranean Sea (Bensoussan et al., 2019). For the Cap de Creus population, 2013 stands out as a period of elevated stress reaching levels comparable to those found in the Columbretes Islands for 2003 and 2021, although no corresponding stress marks were found in the other sites for that year. The *C. caespitosa* population of Montgrí appeared to be the least affected, with a markedly lower proportion of stress marks, hinting again at the potentially enhanced resilience due to higher nutrient concentrations at this site. The variability in the occurrence of stress marks between sites further emphasizes the geographic fluctuation in the occurrence of stress factors for coral growth, such as MHWs, impacting sites with variable strength and not always in all areas at the same time (Garrabou et al., 2022; Martínez et al., 2023).

## 5 CONCLUSIONS

Due to their widespread impact on Mediterranean ecosystems, mass mortalities of benthic organisms have received much attention in recent decades (Garrabou et al., 2009, 2022; Grenier et al., 2023; Marbà et al., 2015). In this context, our results clearly indicate that warming impacts on the Mediterranean corals go beyond mortality events, highlighting the complexity of the responses of these organisms to climate change. This finding is crucial for understanding the long‐term warming impacts on *C. caespitosa*, which until now were primarily related to necrosis events. The results of this study also reveal that water temperature at some sites is nearing levels that could permanently affect growth. Added to recurrent mortality events, this increasing pressure on corals emphasizes the need to consider growth and temperature relationships in assessing long‐term population trajectories. Therefore, our results further underline the vulnerability of *C. caespitosa* populations to climate change. However, the understanding of sublethal responses of this coral to warming remains partial. Consequently, it is essential to gather more empirical information across a broader geographic range, encompassing the divergent environmental conditions in which this coral thrives, and considering other processes such as reproduction. Understanding the interactions between coral growth and environmental parameters, such as nutrient availability and temperature regimes, is crucial to identify the vulnerabilities of corals and foresee how they may respond to future stress events. In turn this information helps devise targeted management and conservation efforts.

## AUTHOR CONTRIBUTIONS

Marina J. Vergotti, Diego K. Kersting and Juan Pablo D'Olivo conceived the ideas behind this study. Marina J. Vergotti and Pol Capdevila carried out the analyses. Diego K. Kersting and Juan Pablo D'Olivo supervised the findings of this work. Thomas C. Brachert and Philipp M. Spreter contributed with guidance, materials and methods to obtain the X‐ray images. Pol Capdevila contributed with guidance and expertise on the statistical methods. Cristina Linares and Joaquim Garrabou contributed with temperature data. Marina J. Vergotti took the lead in writing the manuscript and all coauthors provided critical feedback and helped shape the manuscript.

## CONFLICT OF INTEREST STATEMENT

The authors declare no conflict of interest.

## Supporting information


**Method S1.** Bulk skeletal density calculations from X‐ray images, using coralXDS.
**Method S2.** Nomralization proceedure for the coral grwth data.
**Figure S1.** Example of a transect performed on a corallite X‐ray using Coral XDS (a) and the output plot of skeletal density corresponding to the transect (b). In these plots, the program shows LD bands in white and HD bands in grey. Bellow the plot are indicated the corresponding HD and LD band values for extension (E), calcification (C) and density (D). Are also shown the annual band values (Ann), computed as the sum of HD and LD bands for extension, and the mean of HD and LD bands for calcification and density.
**Figure S2.** Box‐and whisker plots of the average linear extension (a), skeletal density (b), calcification (c), and LD/HD band ratio (d), for each site. Different letters indicate significant differences among sites. Boxes hold 50% of the data, with the bold horizontal line in the box indicating the median of the distribution. Whiskers hold the lower and higher 25% of the data, with the end of each whisker indicating respectively the minimum and the maximum values of the distribution. Dots outside the box‐and‐whisker plot indicate outliers of the distribution.
**Figure S3.** Average annual (a) linear extension, (b) density, and (c) calcification as a function of in situ temperature for the three sites. The black curves display the fit of the generalized additive mixed models (GAMM, in black) for the combined data from all three sites with the 95% confidence interval (in grey). Growth values from individual corallites are indicated by coloured points and are grouped by sites, i.e. Columbretes islands (in blue), Montgrí (in green), and Cap de Creus (in orange).
**Table S1.** All models were tested for normality, dispersion and homoscedasticity using the R package “DHARMa” (Hartig 2018). Q‐Q plots were used to test the normality of the data and residuals dispersion plots to test the homogeneity of the variance and the dispersion of the data.Model diagnostics are presented as follows: (I) Generalized Additive Mixed Models (GAMM) of the growth parameters related to average annual in situ temperature at the regional level, (II) Linear Mixed Models (LMM) relating, at the regional site, the growth parameters with average annual, summer and warmest month in situ temperatures, and the same LMM models applied at the site level for the Columbretes Islands (III.a with in situ temperatures and III.b with SST), Montgrí (IV) and Cap de Creus (V).
**Table S2.** Summary of the LMMs relating linear extension, density and calcification with average annual temperatures (A), average summer temperatures (B) and average temperatures of the warmest month (C), at the regional level, as well as for each site individually. In the case of the Columbretes Islands, SST was also included in the models.

## Data Availability

The data that support the findings of this study is available in the Zenodo online repository https://doi.org/10.5281/zenodo.13710294 (Vergotti et al., 2024).
