# Supplementary material for: Reconstruction of long‐term sublethal effects of warming on a temperate coral in a climate change hotspot
Source: J Anim Ecol. 2024 Nov 19;94(1):125–38. doi: 10.1111/1365-2656.14225 (PMC11730637; doi:10.1111/1365-2656.14225)
Supplement: Supplementary file 1 — Method S1. Bulk skeletal density calculations from X‐ray images, using coralXDS. Method S2. Nomralization proceedure for the coral grwth data. Figure S1. Example of a transect performed on a corallite X‐ray using Coral XDS (a) and the output plot of skeletal density corresponding to the transect (b). In these plots, the program shows LD bands in white and HD bands in grey. Bellow the plot are indicated the corresponding HD and LD band values for extension (E), calcification (C) and density (D). Are also shown the annual band values (Ann), computed as the sum of HD and LD bands for extension, and the mean of HD and LD bands for calcification and density. Figure S2. Box‐and whisker plots of the average linear extension (a), skeletal density (b), calcification (c), and LD/HD band ratio (d), for each site. Different letters indicate significant differences among sites. Boxes hold 50% of the data, with the bold horizontal line in the box indicating the median of the distribution. Whiskers hold the lower and higher 25% of the data, with the end of each whisker indicating respectively the minimum and the maximum values of the distribution. Dots outside the box‐and‐whisker plot indicate outliers of the distribution. Figure S3. Average annual (a) linear extension, (b) density, and (c) calcification as a function of in situ temperature for the three sites. The black curves display the fit of the generalized additive mixed models (GAMM, in black) for the combined data from all three sites with the 95% confidence interval (in grey). Growth values from individual corallites are indicated by coloured points and are grouped by sites, i.e. Columbretes islands (in blue), Montgrí (in green), and Cap de Creus (in orange). Table S1. All models were tested for normality, dispersion and homoscedasticity using the R package “DHARMa” (Hartig 2018). Q‐Q plots were used to test the normality of the data and residuals dispersion plots to test the homogeneity of the variance and the dispersion [file JANE-94-125-s001.docx]

# Supplementary materials

**Method S1**

The coral XDS software measures changes in grey scale values of the X-ray image to calculate the bulk skeletal density of the coral sample. To do this, a linear regression curve is calculated using a standard material of known density to convert relative optical density to absolute density values. For this, an aluminium wedge of 9.9 cm in length, 0.138º in slope, and 2.71 g·cm^-3^ in density was also X-rayed and analysed to produce a calibration curve to be used as a density standard. Plates of aluminium of different thicknesses ranging from 4.0 mm to 5.0 mm (to cover the range in thickness found in the coral samples) were X-rayed to generate background images used to correct for area variations in the beam intensity and reduce uncertainties in the density values. A calibration curve was generated, respectively, for each background image and corresponding thickness. For each corallite, the density standard and background image were selected closest to the average thickness of the corallite, based on 10 thickness measurements along their length. The goodness of fit of the density calibration curves was verified against X-rays of a homogenous shell of *Tridacna* spp*.* of known density (2.93 g·cm^‑3^).

**Reference:**

Spreter PM, Reuter M, Mertz-Kraus R, et al (2022) Calcification response of reef corals to seasonal upwelling in the northern Arabian Sea (Masirah Island, Oman). Biogeosciences 19:3559–3573. https://doi.org/10.5194/bg-19-3559-2022

## Method S2

The unequal length of the corallite samples resulted in a variable sample size per year. In order to equally weigh the growth values of each year in a time series, a normalization procedure was applied as:

$$z_{t}=\left( x_{t}-\overline{x_{c}} \right)+\overline{x_{n}}$$

The mean growth value of each given corallite ($\overline{x_{c}}$) was first calculated over a common period defined between the colonies of a site. This value was then subtracted from the annual growth values of each corallite ($x_{t}$), providing their normalized annual growth values. Finally, the overall average growth value ($\overline{x_{n}}$) was calculated over the common period using all raw data and added to the normalized annual growth values of each corallites, maintaining the units of those values ($z_{t}$).

For each site, the common period was defined by englobing years with more than 35% of the total sample size. In the case of the Columbretes Islands, the newer colonies (collected in October 2022) were quite shorter than the older colonies (collected between August 2014 and 2017). This resulted in a global common period that was not representative of the newer colonies, as only a few samples had bands covering those years. Thus, is was necessary to consider two different common periods, for the newer and the older colonies. The resulting common periods per site are as follows: Columbretes Islands, 2006-2016 and 2016-2021, Montgrí, 2014-2019 and Cap de Creus, 2015-2019.

As the newer colonies and older colonies of the Columbretes Islands had significantly different growth rates, an additional scaling procedure was performed. For this, the common period of both groups was defined as 2011-2017 and the average growth value was calculated for each group over this period. An aliquot of the new corallites was used in this case, selecting only those corallites covering at least half of the period. The resulting offset between the average values calculated for the new colonies and the colonies was then subtracted from the normalized annual growth values of corallites pertaining to the new colonies.

**Reference:**

D’Olivo JP, McCulloch MT, Judd K (2013) Long-term records of coral calcification across the central Great Barrier Reef: assessing the impacts of river runoff and climate change. Coral Reefs 32:999–1012. https://doi.org/10.1007/s00338-013-1071-8


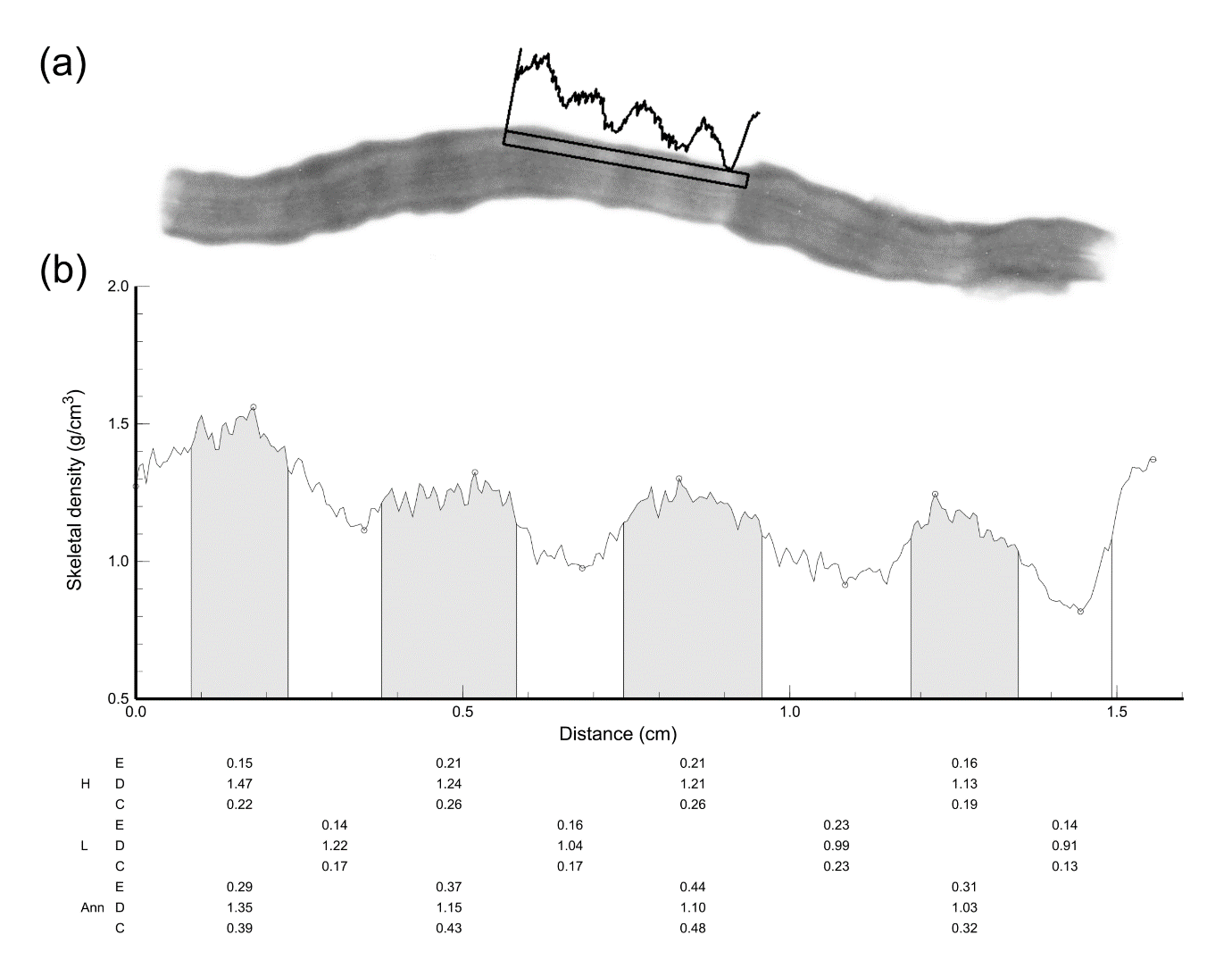


**Figure S1.** Example of a transect performed on a corallite X-ray using Coral XDS (A) and the output plot of skeletal density corresponding to the transect (B). In these plots, the program shows LD bands in white and HD bands in grey. Bellow the plot are indicated the corresponding HD and LD band values for extension (E), calcification (C) and density (D). Are also shown the annual band values (Ann), computed as the sum of HD and LD bands for extension, and the mean of HD and LD bands for calcification and density.


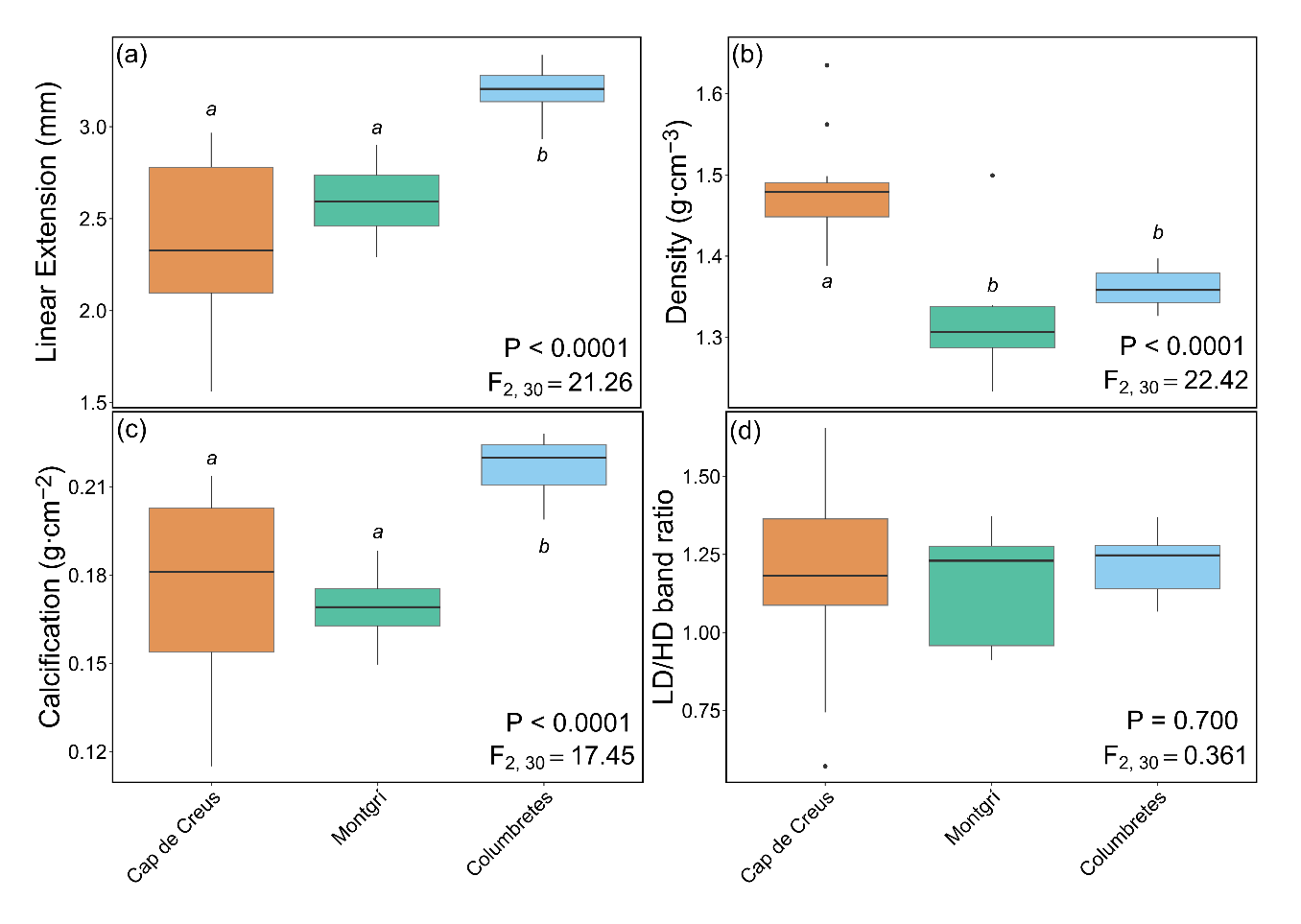


**Figure S2.** Box-and whisker plots of the average linear extension (a), skeletal density (b), calcification (c), and LD/HD band ratio (d), for each site. Different letters indicate significant differences among sites. Boxes hold 50% of the data, with the bold horizontal line in the box indicating the median of the distribution. Whiskers hold the lower and higher 25% of the data, with the end of each whisker indicating respectively the minimum and the maximum values of the distribution. Dots outside the box-and-whisker plot indicate outliers of the distribution.


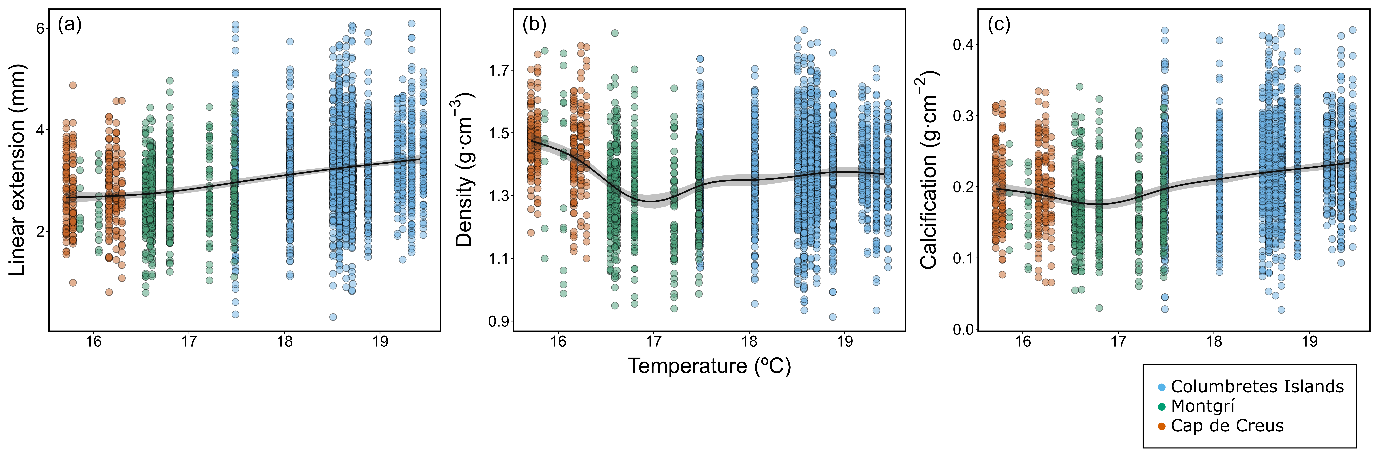


**Figure S3.** Average annual a) linear extension, b) density, and c) calcification as a function of in situ temperature for the three sites. The black curves display the fit of the generalized additive mixed models (GAMM, in black) for the combined data from all three sites with the 95% confidence interval (in grey). Growth values from individual corallites are indicated by coloured points and are grouped by sites, i.e. Columbretes islands (in blue), Montgrí (in green), and Cap de Creus (in orange).

**Table S1.** All models were tested for normality, dispersion and homoscedasticity using the R package “DHARMa” (Hartig 2018). Q-Q plots were used to test the normality of the data and residuals dispersion plots to test the homogeneity of the variance and the dispersion of the data. Model diagnostics are presented as follows: I) Generalized Additive Mixed Models (GAMM) of the growth parameters related to average annual *in situ* temperature at the regional level, II) Linear Mixed Models (LMM) relating, at the regional site, the growth parameters with average annual, summer and warmest month *in situ* temperatures, and the same LMM models applied at the site level for the Columbretes Islands (III.a with *in situ* temperatures and III.b with SST), Montgrí (IV) and Cap de Creus (V).

**I) GAMM (annual *in situ* temperature)**

**Linear extension Density Calcification**

**
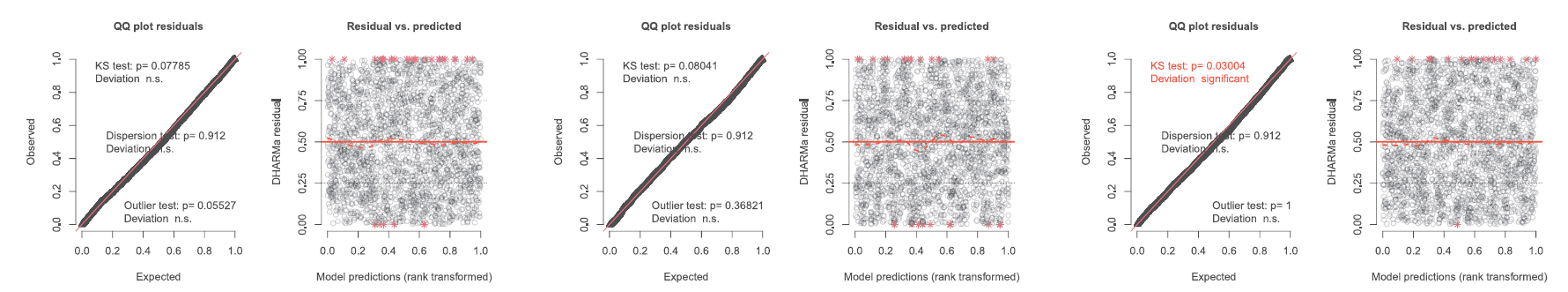
**

**II) LMM at the regional scale**

**Annual temperature**

**Linear extension Density Calcification**

**
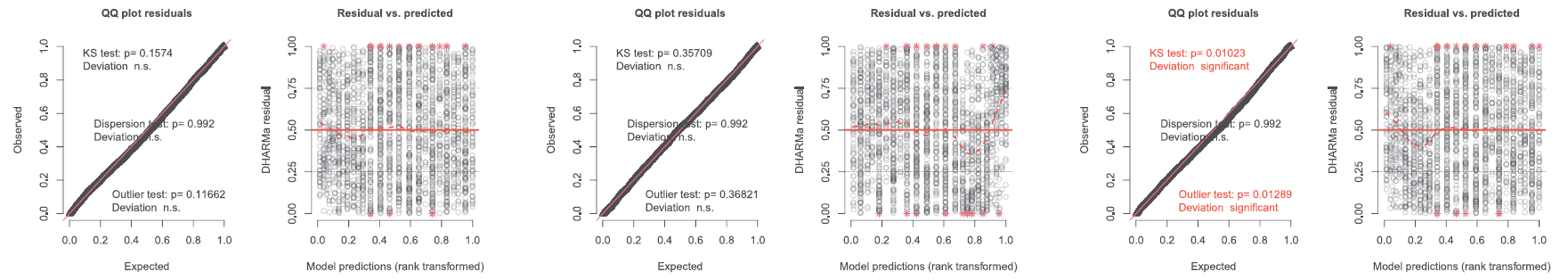
**

**Summer temperature**

**Linear extension Density Calcification**

**
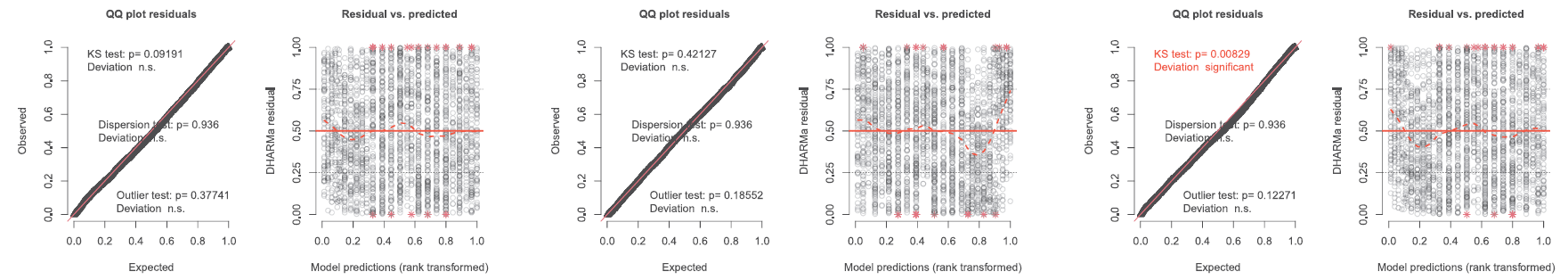
**

**Temperature of the warmest month**

**Linear extension Density Calcification**

**
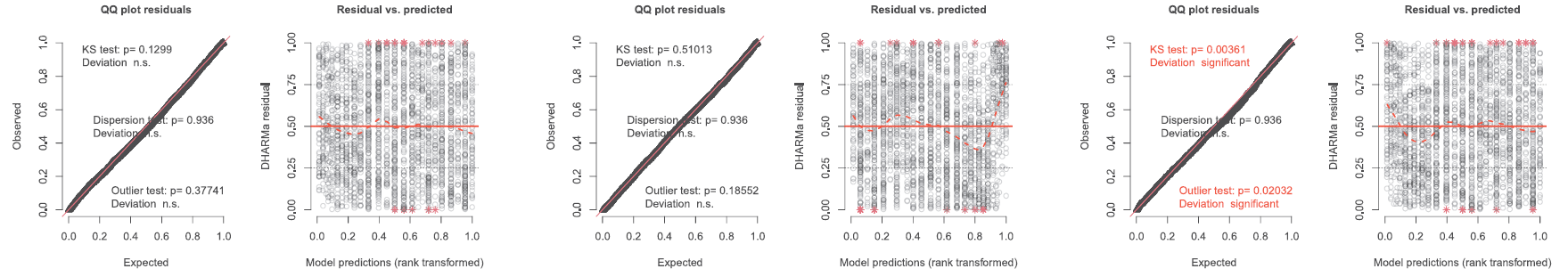
**

**III.a) LME at site level: Columbretes Islands (*in situ* temperatures)**

**Annual temperature**

**Linear extension Density Calcification**

**
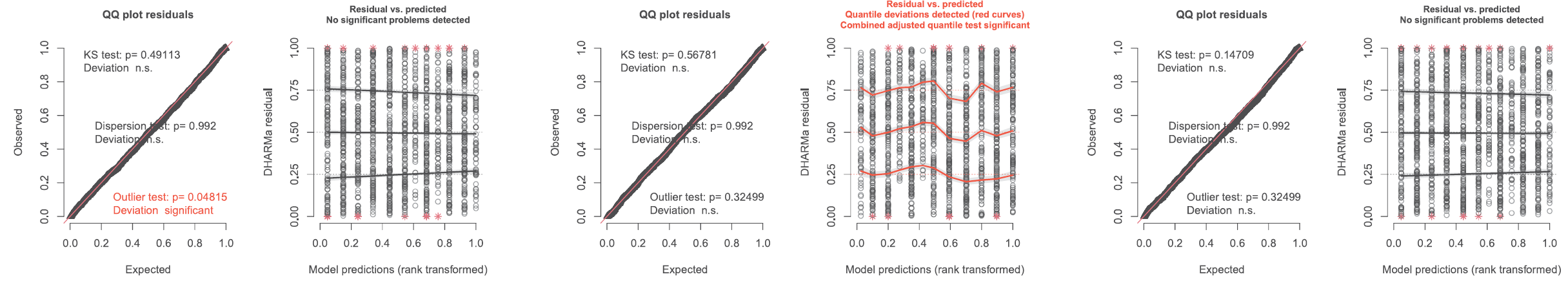
**

**Summer temperature**

**Linear extension Density Calcification**

**
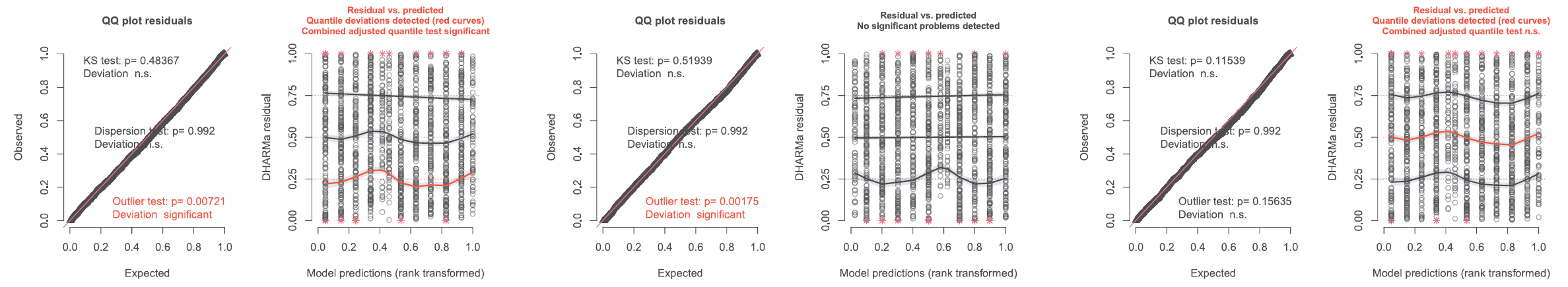
**

**Temperature of the warmest month**

**Linear extension Density Calcification**

**
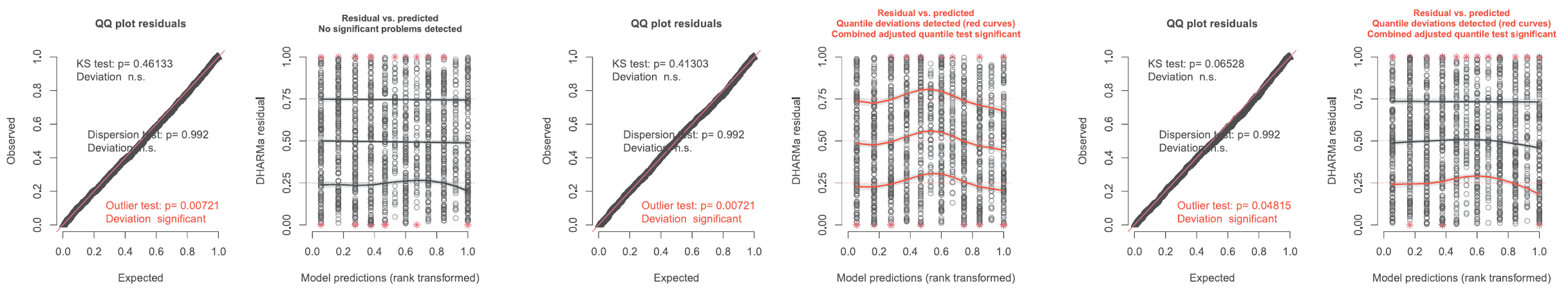
**

**III.b) LME at site level: Columbretes Islands (Sea Surface Temperatures)**

**Annual temperature**

**Linear extension Density Calcification**

**
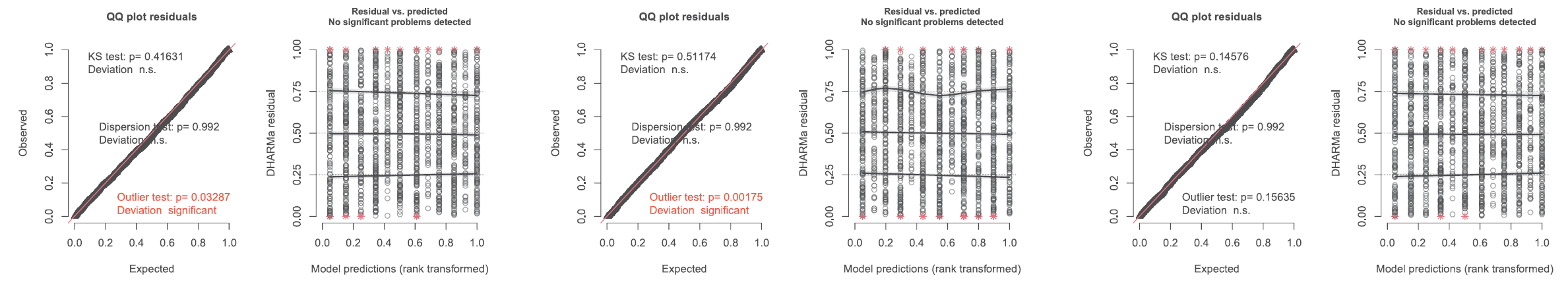
**

**Summer temperature**

**Linear extension Density Calcification**

**
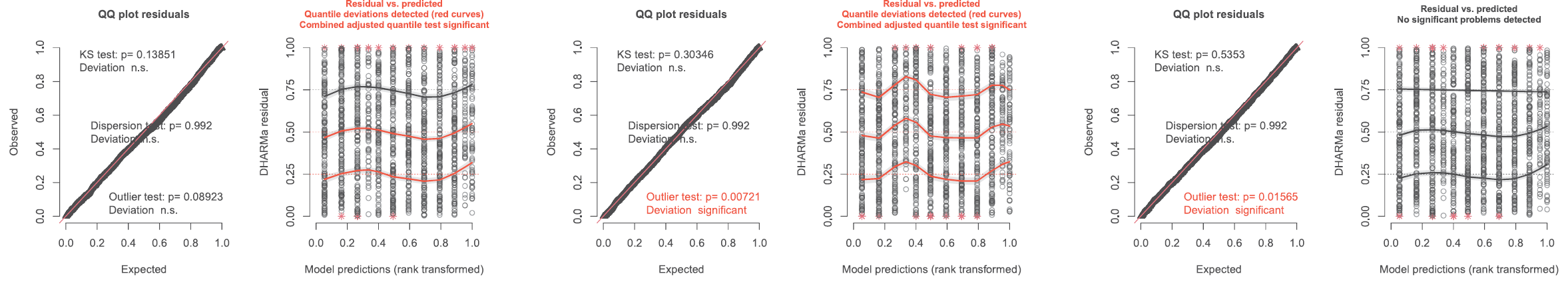
**

**Temperature of the warmest month**

**Linear extension Density Calcification**

**
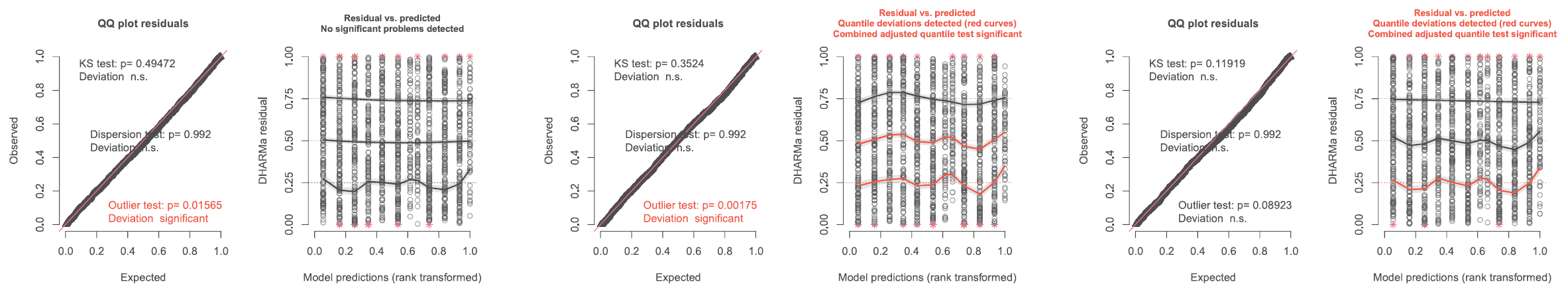
**

**IV) LME at site level: Montgrí**

**Annual temperature**

**Linear extension Density Calcification**

**
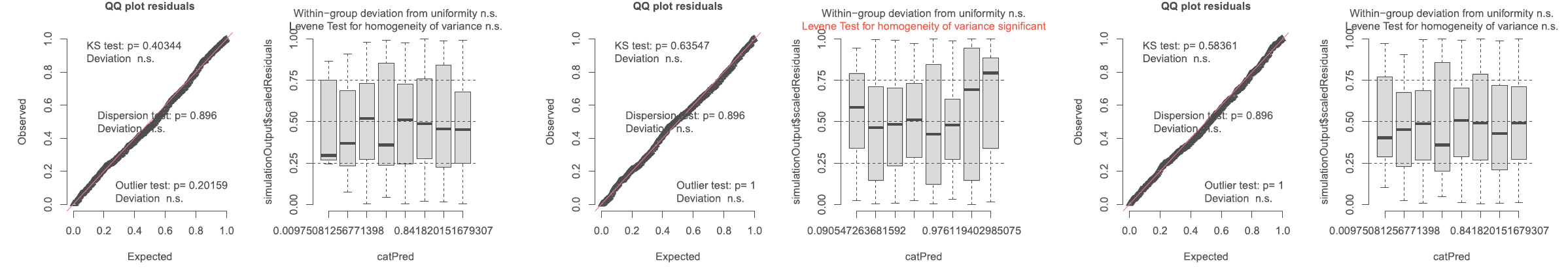
**

**Summer temperature**

**Linear extension Density Calcification**

**
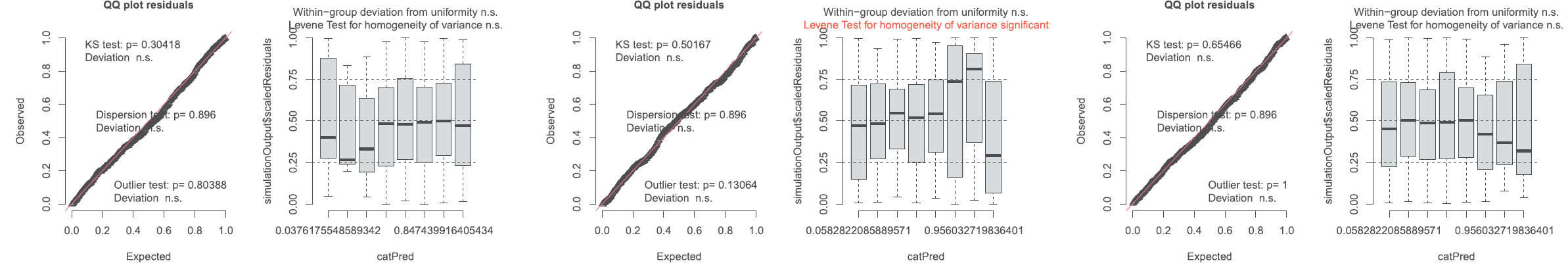
**

**Temperature of the warmest month**

**Linear extension Density Calcification**

**
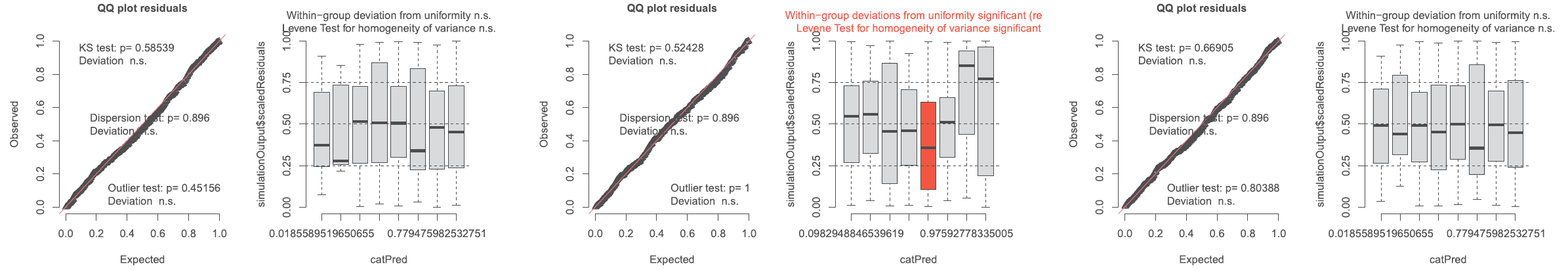
**

**V) LME at site level: Cap de Creus**

**Annual temperature**

**Linear extension Density Calcification**

**
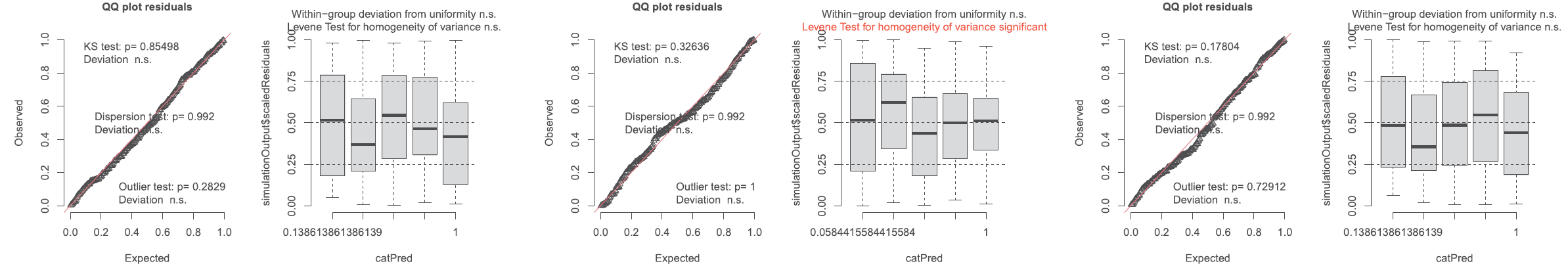
**

**Summer temperature**

**Linear extension Density Calcification**

**
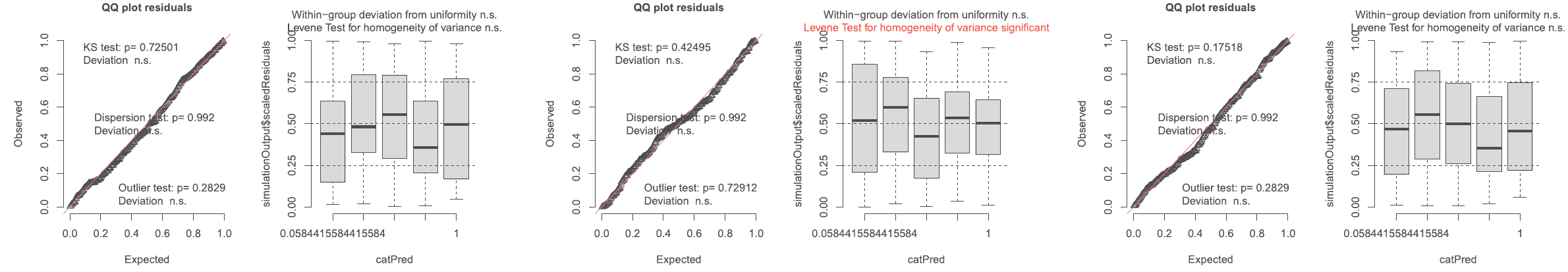
**

**Temperature of the warmest month**

**Linear extension Density Calcification**

**
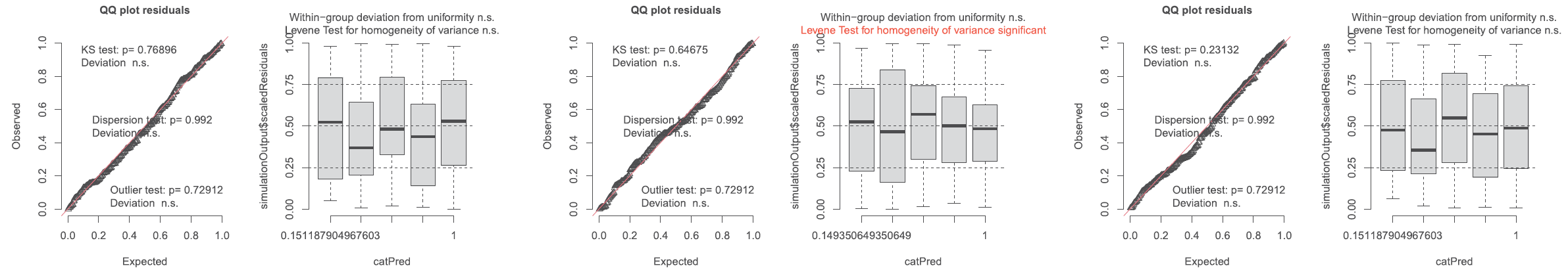
**

**Reference**

Hartig F (2018) DHARMa: residual diagnostics for hierarchical (multi-level/mixed) regression models. R Package Version 020

| ***Table S2.*** *Summary of the* *LMMs relating linear extension, density and calcification with average annual temperatures (A), average summer temperatures (B) and average temperatures of the warmest month (C), at the regional level, as well as for each site individually. In the case of the Columbretes Islands, SST was also included in the models.*   \| *a. Average annual temperature* \| \| \| \| \| \| \| \| \| \| \| \| --- \| --- \| --- \| --- \| --- \| --- \| --- \| --- \| --- \| --- \| --- \| \|  \| All sites \| \| Columbretes Islands \| \| \| \| Montgrí \| \| Cap de Creus \| \| \|  \| In situ \| \| SST \| \| In situ \| \| In situ \| \| In situ \| \| \|  \| β \| p-Value \| β \| p-Value \| β \| p-Value \| β \| p-Value \| β \| p-Value \| \| Extension \| **0.229** \| **<0.001** \| **0.126** \| **0.006** \| **0.126** \| **0.002** \| **0.162** \| **0.043** \| 0.099 \| 0.596 \| \| Density \| -0.004 \| 0.097 \| -0.001 \| 0.908 \| -0.001 \| 0.913 \| **-0.060** \| **<0.001** \| **-0.067** \| **0.033** \| \| Calcification \| **0.016** \| **<0.001** \| **0.009** \| **0.007** \| **0.009** \| **0.004** \| 0.002 \| 0.666 \| 0.003 \| 0.824 \| \| *b. Average Summer temperature* \| \| \| \| \| \| \| \| \| \| \| \|  \| All sites \| \| Columbretes Islands \| \| \| \| Montgrí \| \| Cap de Creus \| \| \|  \| In situ \| \| SST \| \| In situ 15m \| \| In situ \| \| In situ \| \| \|  \| β \| p-Value \| β \| p-Value \| β \| p-Value \| β \| p-Value \| β \| p-Value \| \| Extension \| **0.149** \| **<0.001** \| 0.096 \| 0.181 \| **0.094** \| **0.019** \| 0.107 \| 0.317 \| -0.007 \| 0.967 \| \| Density \| -0.001 \| 0.559 \| **0.024** \| **0.020** \| <-0.001 \| 0.991 \| **-0.069** \| **0.001** \| **-0.061** \| **0.043** \| \| Calcification \| **0.010** \| **<0.001** \| **0.010** \| **0.031** \| **0.006** \| **0.024** \| -0.005 \| 0.508 \| -0.006 \| 0.676 \| \| *c. Average temperature of the warmest month* \| \| \| \| \| \| \| \| \| \| \| \|  \| All sites \| \| Columbretes Islands \| \| \| \| Montgrí \| \| Cap de Creus \| \| \|  \| In situ \| \| SST \| \| In situ 15m \| \| In situ \| \| In situ \| \| \|  \| β \| p-Value \| β \| p-Value \| β \| p-Value \| β \| p-Value \| β \| p-Value \| \| Extension \| **0.101** \| **<0.001** \| 0.018 \| 0.690 \| 0.062 \| 0.067 \| 0.107 \| 0.125 \| 0.057 \| 0.508 \| \| Density \| <-0.001 \| 0.790 \| **0.027** \| **<0.001** \| **0.014** \| **0.011** \| -0.019 \| 0.169 \| **-0.049** \| **0.001** \| \| Calcification \| **0.007** \| **<0.001** \| 0.006 \| 0.070 \| **0.006** \| **0.004** \| 0.005 \| 0.226 \| <-0.001 \| 0.963 \| \|  \| \| \| \| \| \| \| \| \| \| \| |
| --- | --- | --- | --- | --- | --- | --- | --- | --- | --- | --- | --- | --- | --- | --- | --- | --- | --- | --- | --- | --- | --- | --- | --- | --- | --- | --- | --- | --- | --- | --- | --- | --- | --- | --- | --- | --- | --- | --- | --- | --- | --- | --- | --- | --- | --- | --- | --- | --- | --- | --- | --- | --- | --- | --- | --- | --- | --- | --- | --- | --- | --- | --- | --- | --- | --- | --- | --- | --- | --- | --- | --- | --- | --- | --- | --- | --- | --- | --- | --- | --- | --- | --- | --- | --- | --- | --- | --- | --- | --- | --- | --- | --- | --- | --- | --- | --- | --- | --- | --- | --- | --- | --- | --- | --- | --- | --- | --- | --- | --- | --- | --- | --- | --- | --- | --- | --- | --- | --- | --- | --- | --- | --- | --- | --- | --- | --- | --- | --- | --- | --- | --- | --- | --- | --- | --- | --- | --- | --- | --- | --- | --- | --- | --- | --- | --- | --- | --- | --- | --- | --- | --- | --- | --- | --- | --- | --- | --- | --- | --- | --- | --- | --- | --- | --- | --- | --- | --- | --- | --- | --- | --- | --- | --- | --- | --- | --- | --- | --- | --- | --- | --- | --- | --- | --- | --- | --- | --- | --- | --- | --- | --- | --- | --- | --- | --- | --- | --- | --- | --- | --- | --- | --- | --- | --- | --- | --- | --- | --- | --- | --- | --- | --- | --- | --- | --- | --- | --- | --- | --- | --- | --- | --- | --- | --- | --- | --- | --- | --- | --- | --- | --- | --- | --- | --- | --- | --- | --- | --- | --- | --- | --- | --- |
